# Supplementary material for: Assessment of the Interferon-Lambda-3 Polymorphism in the Antibody Response to COVID-19 in Older Adults Seropositive for CMV
Source: Vaccines (Basel). 2023 Feb 18;11(2):480. doi: 10.3390/vaccines11020480 (PMC9963200; doi:10.3390/vaccines11020480)
Supplement: Supplementary file 1 [file vaccines-11-00480-s001.zip › vaccines-2192071-supplementary.pdf]

Table S1. Results of neutralizing antibodies assessment for two different SARS-CoV-2 variants (Wuhan and Delta) in each volunteer enrolled in the present study on three different time points: MO (micro-outbreak), CURE, and VAC (vaccination). The range applied was 1:20 to 1:2560 and the values below 20 (<20) were considered negatives.

	SARS-CoV-2 variant					
	**VNT B (Wuhan)**			**VNT Delta**		
Volunteer number	MO	CURE)	VAC	MO	CURE)	VAC
1	20	40	20	20	20	20
2	<20	<20	20	<20	<20	20
3	<20	<20	<20	<20	<20	<20
4	<20	<20	80	<20	<20	20
5	<20	<20	<20	<20	<20	<20
6	80	40	1280	<20	<20	≥2560
7	<20	<20	-	<20	<20	-
8	<20	<20	<20	<20	<20	<20
9	<20	<20	<20	<20	<20	<20
10	<20	<20	<20	<20	<20	<20
11	<20	<20	<20	<20	<20	<20
12	<20	320	≥2560	<20	80	1280
13	<20	<20	<20	<20	<20	<20
14	<20	<20	<20	<20	<20	<20
15	<20	<20	-	<20	<20	-
16	-	<20	80	-	<20	80
17	640	160	≥2560	320	80	640
18	<20	320	1280	<20	80	640
19	<20	320	160	<20	80	40
20	<20	160	≥2560	<20	80	1280
21	<20	-	<20	<20	-	<20
22	<20	<20	320	<20	<20	320
23	<20	40	40	<20	<20	<20
24	<20	<20	-	<20	<20	-
25	<20	40	20	<20	20	20
